# A survey of photogeochemistry

**DOI:** 10.1186/s12932-017-0039-y

**Published:** 2017-02-10

**Authors:** Timothy A. Doane

**Affiliations:** Department of Land, Air and Water Resources, University of California, Davis, Davis, CA 95616-5270 USA

**Keywords:** Atmosphere, Minerals, Natural photoreactions, Photocatalysis, Photochemistry, Soil, Surface geochemistry, Water

## Abstract

The participation of sunlight in the natural chemistry of the earth is presented as a unique field of study, from historical observations to prospects for future inquiry. A compilation of known reactions shows the extent of light-driven interactions between naturally occurring components of land, air, and water, and provides the backdrop for an outline of the mechanisms of these phenomena. Catalyzed reactions, uncatalyzed reactions, direct processes, and indirect processes all operate in natural photochemical transformations, many of which are analogous to well-known biological reactions. By overlaying photochemistry and surface geochemistry, complementary approaches can be adopted to identify natural photochemical reactions and discern their significance in the environment.

## Background

Photogeochemistry has been defined as the photochemistry of Earth-abundant minerals in shaping biogeochemistry [1], and this can be extended to the entire interface between photochemistry and geochemistry to include any chemical reaction induced by sunlight among naturally occurring substances. The term has been used previously on only several other isolated occasions [2, 3], but if existing research is surveyed for studies that fit this definition, an appreciable body of knowledge emerges.

**Fig. 1:**
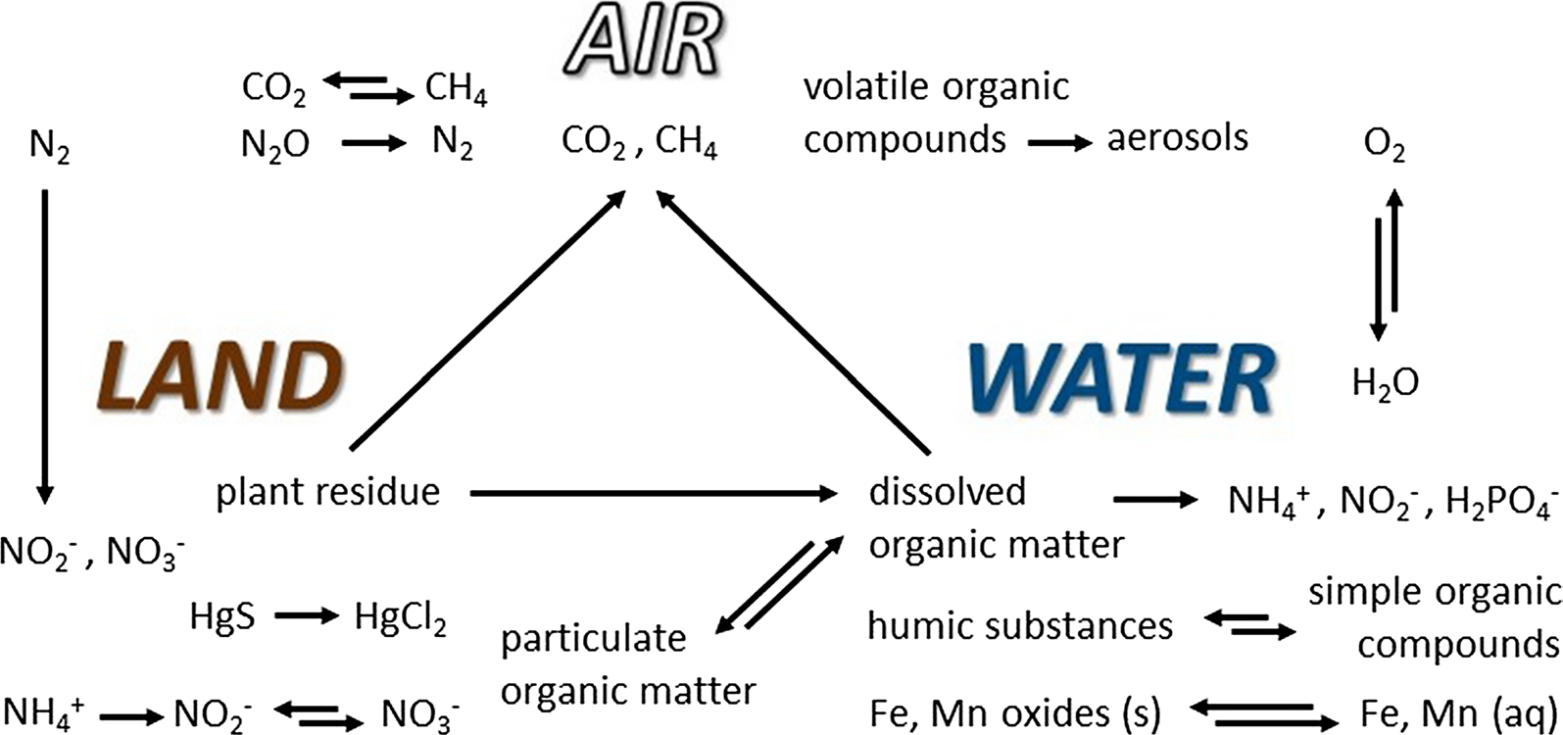
Photogeochemistry is the study of sunlight-induced chemical reactions among substances that are found naturally on Earth’s surface and intermingle across its domains. Examples of photochemical reactions are shown that occur in the basic domains of land, air, and water. Reaction details and references can be found in Table 1

The context of a photogeochemical reaction is implicitly the surface of the earth, since that is where sunlight is available (ignoring other sources of light such as bioluminescence). Reactions may occur among constituents of land such as minerals, plant residue, and the organic and inorganic components of soil; constituents of surface water such as sediment and dissolved organic matter; and constituents of the atmospheric boundary layer directly influenced by contact with land or water, such as organic aerosols, mineral aerosols, and gases. Figure 1 shows some examples of photochemical reactions among these substances. Sunlight penetrates up to approximately 0.3 mm in soils and particulate minerals, depending on the wavelength of light and the nature of the particles [4], and many meters in clear water, depending on the concentration of light-absorbing molecules [5, 6]. Light of wavelengths less than about 290 nm is completely absorbed by the present atmosphere and therefore does not reach Earth’s surface [7, 8].

Photogeochemistry describes photochemical reactions on Earth that are not facilitated by living organisms. The reactions that comprise photosynthesis in plants and other organisms, for example, are not included, since the physiochemical context for these reactions is installed by the organism, and must be maintained in order for the reactions to continue (the photoreactions cease if the organism dies). However, if a certain substance is produced by an organism, and the organism dies but the substance remains (e.g., plant residue or biogenic mineral precipitates), photoreactions involving this substance still contribute to photogeochemistry.

## History

The most famous example of a photochemical reaction involving natural compounds is the production of indigoid dyes from the secretions of marine mollusks, known since antiquity [9]; the role of sunlight was emphasized in a study by William Cole in 1685 [10]. The development of modern photochemistry in general was fostered by similar adventitious observations of the effect of sunlight on natural compounds. For example, Hyde Wollaston in 1811 [11] observed that guaiac, a tree resin, rapidly turned green in the air when exposed to sunlight (due to photooxidation). Natural photodegradation was also known, as described by Berzelius in 1829 [12]: “Light fades and destroys the majority of plant colorants. Every day we see that of the sun weakening the dyes of our fabrics”. This phenomenon was also mentioned by John William Draper in 1845 [13]. Georges Witz in 1883 described the degradation of cellulose by sunlight, remarking on the influence of air and moisture, and further noted that degradation was greatly accelerated by ferric oxide [14]. By the end of the 19th century, photodegradation of organic matter in natural waters was recognized as a universal phenomenon [15]. In addition to degradation, other light-induced transformations were also recorded. Louis Pasteur described how a dark-colored material is produced in cinchona bark under the influence of sunlight, an observation that he confirmed in the laboratory with specific compounds [16], and Hermann Trommsdorff [17] and Karl Fritzsche [18] were also among those who observed changes in natural organic substances when they were illuminated. Many inorganic substances were also known to change (e.g., in color or crystal structure) upon exposure to light [13]. For example, since 1881 it has been known that zinc sulfide, normally white, becomes dark when exposed to sunlight [19]; John Cawley remarked that “I have prepared pigments so sensitive as to be turned almost black when exposed to bright sunlight for one or two minutes” [20]. Investigation of the light-induced reactions of this compound [21], which occurs as a natural mineral, provided some additional empirical contributions to photochemistry and the “photochemical metallurgy” of zinc, and its photocatalytic properties are still studied at present [22, 23]. Many natural inorganic compounds used throughout the ages as pigments in painting also slowly degrade by exposure to sunlight; artists like Van Gogh were aware of this [24]. Some of these compounds, such as mercury(II) sulfide, undergo a number of light-mediated reactions [25] which are environmentally relevant.

Around the time of these and other observations, experiments increased in an effort to reproduce natural processes. The hypothesis of von Baeyer in 1870 [26], in which formaldehyde was proposed to be the initial product of plant photosynthesis followed by polymerization into sugars, inspired numerous attempts to obtain formaldehyde from carbon dioxide and water. For example, the formation of lower uranium oxides was observed upon irradiation of a solution of uranium acetate and carbon dioxide, implying the formation of a reducing agent assumed to be formaldehyde [27]. Some experiments included reducing agents such as hydrogen gas [28], and others reportedly detected formaldehyde and other products in the absence of additives [29, 30], suggesting that reducing power was produced from the decomposition of water during exposure to light. In addition to this main focus on the synthesis of formaldehyde and simple sugars, other light-driven reactions were occasionally noted, such as the decomposition of formaldehyde and subsequent release of methane [28]. Many experiments explored the effect of a catalyst in converting light energy into chemical energy; some effective “transformers” (as they were sometimes called) were similar to naturally occurring minerals, including iron(III) oxide or colloidal iron(III) hydroxide [30–32], zinc oxide [33], and cobalt, copper, nickel, and iron carbonates [30, 33]. By this time, interest had spread to other light-induced reactions involving naturally occurring materials. These studies sometimes reported photoreactions analogous to biological processes, such as oxidation of simple carbon compounds [34] or nitrification in soil [35].

## Overview of photogeochemical reactions

Table 1 presents a selection of documented photochemical reactions (with light >290 nm) among naturally occurring substances, ranging from general reactions such as mineralization of organic matter to specific reactions such as methylation and demethylation of mercury. This compilation is by no means exhaustive, either in reactions or references, but illustrates the general scope and diversity of abiotic photochemical reactions that may occur at the surface of the earth.

**Table 1:** Photochemical reactions of naturally occurring substances

Reaction	Descriptor	Facilitators	References
*Carbon compounds*			
Plant material → CO_2_	(Oxidative) photochemical decomposition (mineralization)		[124, 125, 166 (CO_2_ implied), 167]
Plant material (litter and living foliage) → CO	Photochemical decomposition (mineralization)		[125, 168–171]
plant material (litter) → CH_4_	(Reductive) photochemical decomposition (mineralization/methanification)		[172–174]
Plant material (foliage) → CH_4_	(Reductive) photochemical mineralization		[171, 173–176]
Plant material → ethane, ethene, propene, butane, other hydrocarbons	(Reductive) photochemical decomposition		[171, 177]
Plant material → dissolved organic matter	Photochemical decomposition + dissolution		[115]
Plant material → biologically more labile compounds	Photochemical priming (encouraging subsequent biotic decomposition)		[136, 178, 179]
Solid organic matter → CO_2_	(Oxidative) photochemical decomposition (mineralization)	Sand	[180]
Soil organic matter → CH_4_	(Reductive) photochemical decomposition (mineralization/methanification)		[181]
Sorbed or particulate organic matter → dissolved organic matter	Photochemical dissolution		[115, 182, 183]
Dissolved and colloidal organic matter → amino acids	Photochemical decomposition (depolymerization)		[184]
(Nonspecific) decomposition of dissolved organic matter	Photochemical decomposition	No facilitator Aqueous and solid iron(III) species	[70, 109, 185–187]
Dissolved organic matter → CO	(Oxidative) photochemical decomposition (mineralization)		[188 *–* 191]
Dissolved organic matter → CO_2_	(Oxidative) photochemical decomposition (mineralization)	No facilitator TiO_2_	[190, 192–194]
Dissolved organic matter → CH_4_	(Reductive) photochemical decomposition (mineralization/methanification)		[195]
Dissolved organic matter → biologically more labile compounds	Photochemical priming (encouraging subsequent biotic decomposition)		[134, 135, 196]
Humic substances → humic substances with increased carboxylic acid content	photochemical oxidation + acidification		[185]
Dissolved organic matter → organic matter with increased aliphatic content	Photochemical aliphatization		[63, 193]
Humic substances → small carboxylic acids; increased hydrophobicity of remaining organic matter	photochemical decomposition + acidification		[135, 186]
Humic substances → simple carbonyl compounds (e.g., formaldehyde, acetone, pyruvate)	Photochemical decomposition		[189, 197]
Dissolved organic matter → condensed aromatic structures (soluble and particulate)	Photochemical condensation		[193]
Carbohydrates and lipids → oxidized products	Photochemical oxidation	With and without ZnO	[198]
(Nonspecific) decomposition of cellulose	Photochemical decomposition	No facilitator Organic dyes Fe(III) compounds, ZnO, ZnS, TiO_2_	[14, 50, 96, 97, 199]
Cellulose → less polymerized cellulose with increased carbonyl and carboxyl content	Photochemical depolymerization + oxidation		[96, 200]
(Nonspecific) decomposition of chitosan	Photochemical decomposition		[201]
(Nonspecific) decomposition of wool	Photochemical decomposition		[99]
(Nonspecific) decomposition of lignin	Photochemical decomposition	No facilitator TiO_2_	[98, 202, 203]
Lignin → CH_4_, ethane	(Reductive) photochemical decomposition		[204]
Lignin → quinones	(Oxidative) photochemical decomposition		[99, 204, 205]
Lignin → aromatic and aliphatic aldehydes	(Oxidative) photochemical decomposition		[206]
Proteins → larger, aggregated proteins e.g., via intermolecular tyrosine dimerization	Photochemical crosslinking		[207]
Unconjugated unsaturated lipids → conjugated unsaturated lipids + insoluble material	Photochemical isomerization, condensation	Observed in seawater	[208]
Polyunsaturated lipids → humic substances (*proposed reaction*)	(Oxidative) photochemical crosslinking		[209]
Fatty acids → CO_2_, alkenes, aldehydes, ketones, fatty acid dimers	Photochemical oxidation, cleavage, dimerization	No facilitator TiO_2_	[210, 211]
Hydrocarbons e.g., ethane, ethene, propane, butane, paraffin → CO_2_	Photochemical oxidation	TiO_2_	[211, 212]
Long-chain alkanes → ketones, alcohols, acids	Photochemical oxidation	Naphthol, xanthone, anthraquinone	[101]
Dienes + NO_x_ → carboxylic acids	Photochemical oxidation		[213]
Aromatic compounds + NO_x_, NO_2_^−^, or NO_3_^−^ → nitrated aromatic compounds	Photochemical nitration	No facilitator TiO_2_, Fe_2_O_3_	[214–218]
(Nonspecific) decomposition of polycyclic aromatic hydrocarbons	Photochemical decomposition	No facilitator Algae (live or dead) TiO_2_	[138–140, 219]
Polycyclic aromatic hydrocarbons → quinones	Photochemical oxidation	Al_2_O_3_	[78]
Condensed aromatic compounds (dissolved black carbon) → nonspecific products, CO_2_	(Oxidative) photochemical decomposition		[63, 220, 221]
Soot → oxygen-containing species	Photochemical oxidation		[222]
Crude oil → CO_2_	Photochemical oxidation (mineralization)	Sand containing magnetite and ilmenite	[223]
Amino acids → CO_2_	Photochemical oxidation (mineralization)	Cu(II) (aq)	[224, 225]
Amino acids and peptides → smaller carboxylic acids, amines, and amides, NH_3_, CO_2_	(Oxidative) photochemical decomposition, mineralization		[226]
Lysine → pipecolinic acid ornithine → proline	Photochemical cyclization	HgS, ZnS, CdS	[227, 228]
Phenolic ketones and aldehydes → brown carbon	Photochemical oxidation, oligomerization		[155]
Phenol → hydroquinone, catechol → further oxidation products, CO_2_	Photochemical oxidation	Fe_2_O_3_, TiO_2_	[211, 229, 230]
Decomposition of aqueous phenol, naphthol, methylphenols, methoxyphenols, anilines	Photochemical oxidation	Humic and fulvic acids, flavins Algae (live or dead)	[219, 231, 232]
Phenols → phenol dimers	Photochemical coupling/dimerization	Fe(III) (aq)	[102]
Phenols → quinones, naphthols, aminonaphthols → naphthoquinones	Photochemical oxidation	No facilitator NO_3_^−^	[217, 233, 234]
Quinones → quinone dimers	Photochemical coupling/dimerization		[235, 236]
Quinones + benzocyclic olefins → addition products	Photochemical coupling		[237]
Ketones → carboxylic acids	Photochemical cleavage + acidification		[238 *–* 240]
Ketones → CH_4_, ethane	photochemical reduction		[174, 240]
Aromatic ketones → condensed aromatic ring systems	Photochemical condensation		[241]
Vicinal diols → ketones, aldehydes, carboxylic acids	Photochemical cleavage + oxidation	Fe(III) porphyrins	[242]
Cinnamic acid → cinnamic acid dimer	Photochemical coupling/dimerization		[243]
Acetic acid → CH_4_ + CO_2_	Photochemical disproportionation/dismutation	TiO_2_; α-Fe_2_O_3_; Fe_2_O_3_ on montmorillonite (in the absence of O_2_); TiO_2_, Fe_2_O_3_, SrTiO_3_ plus an electron acceptor	[121, 122, 244]
Acetic acid → CO_2_, CH_4_, ethane; methanol, ethanol, propionic acid, other products	Various	α-Fe_2_O_3_; TiO_2_, Fe_2_O_3_, SrTiO_3_, WO_3_ plus an electron acceptor	[122, 211, 244]
Acetate, terpenes + O_2_ → organic (hydro)peroxides	Photochemical peroxidation	No facilitator ZnO, organic sensitizers	[245–247]
Unsaturated lipids + O_2_ → lipid hydroperoxides	Photochemical peroxidation	Chlorophyll	[248, 249]
Propionic acid → ethane + CO_2_ Butyric acid → propane + CO_2_ Salicylic acid → phenol + CO_2_	Photochemical decarboxylation	Fe_2_O_3_ alone or on montmorillonite Algae (live or dead)	[122, 250]
Lactic acid → pyruvic acid + H_2_	Photochemical oxidation + dehydrogenation	ZnS	[251]
Lactic acid → acetaldehyde + CO_2_	(Oxidative) photochemical decarboxylation	Aqueous Cu(II) and Fe(III)	[251, 252]
Glucose → CO_2_	Photochemical oxidation	TiO_2_	[211]
Oxalic acid → CO_2_	Photochemical oxidation	TiO_2_, sand, ash, α-Fe_2_O_3_, γ-Fe_2_O_3_, α-FeOOH, β-FeOOH, γ-FeOOH, δ-FeOOH	[71, 211, 253, 254]
Tartaric, citric, oxalic, malonic acids → oxidized products	Photochemical oxidation	Ferritin	[255]
Pyruvic acid → pyruvic acid oligomers	Photochemical oligomerization		[256]
Salicylic acid → humic-like substances	Photochemical condensation	Accelerated in the presence of algae	[250]
Syringic acid and other methoxybenzoic acids → methanol	Photochemical decomposition		[257]
Syringic acid and related compounds + Cl^−^ → CH_3_Cl	Photochemical decomposition + chlorination		[257]
Methanol → ethylene glycol + H_2_ Ethanol → butane-2,3-diol + H_2_	Photochemical coupling + dehydrogenation	ZnS in the absence of air	[258]
Isoprene → methylthreitol and methylerythritol (aerosols)	Photochemical oxidation		[259]
(Specific) plant compounds → compounds toxic to other organisms	Phototoxicity		[260, 261]
CO_2_ → CO, HCOOH, HCHO, CH_3_OH, CH_4_	Photochemical reduction (one-carbon products)	Fe(III) oxides, FeCO_3_, NiCO_3_, CoCO_3_, CuCO_3_, Mn(II) (aq), ZnO, TiO_2_, ZnS, CdS, ZrO_2_, WO_3_, CaFe_2_O_4_, BiVO_4_, hydrous Cu_2_O, transition metal ions and oxides in zeolites	[30, 31, 33, 262–268]
CO_2_ + H_2_ → CH_4_	Photochemical reduction	α-Fe_2_O_3_ and Zn-Fe oxide in the presence of water, NiO	[269, 270]
CO_2_ + H_2_ → CO, HCOOH, CH_3_OH	Photochemical reduction	α-Fe_2_O_3_ and Zn-Fe oxide in the presence of water	[269]
CO_2_ → HCOOH	Photochemical reduction	Porphyrins, phthalocyanines Elemental Cu on silicate rocks such as granite and shale	[271, 272]
CO_2_ → ethanol CO_2_ → ethane, ethene, propane, propene CO_2_ → tartaric, glyoxylic, oxalic acids	Photochemical reduction (products with more than one carbon)	SiC, ZnS, BiVO_4_, montmorillonite-modified TiO_2_	[273–277]
CH_4_ → HCOOH CH_4_ → CO, CO_2_	Photochemical oxidation	TiO_2_	[211, 278]
CH_4_ → ethane + H_2_	Photochemical coupling + dehydrogenation	SiO_2_-Al_2_O_3_-TiO_2_	[279]
*Nitrogen compounds*			
Plant foliage → NO_x_			[280]
Plant foliage → N_2_O			[281]
Particulate organic N → dissolved organic N and NH_4_^+^	Photochemical decomposition (dissolution + mineralization)		[115]
Dissolved organic N → biologically more labile N	Photochemical priming		[282]
Amino acids and other organic N (including biologically recalcitrant organic N) → NH_4_^+^	Photochemical decomposition (mineralization/ammonification)	No facilitator Organic matter, Fe_2_O_3_, soil	[132, 184, 193, 194, 283–286]
Humic substances → NO_2_^−^	(Oxidative) photochemical decomposition (mineralization)		[104, 287]
NH_3_ → NO_2_^−^ NH_3_ → NO_3_^−^	Photochemical oxidation (nitrification)	TiO_2_, ZnO, Al_2_O_3_, SiO_2_, MnO_2_, soil Observed in seawater	[288 *–* 290]
NH_3_ → N_2_O, N_2_	Photochemical oxidation	TiO_2_	[290, 291]
NH_4_^+^ + NO_2_^−^ → N_2_ urea, protein → [NH_4_NO_2_] → N_2_	Photochemical oxidation + reduction (denitrification)	TiO_2_, ZnO, Fe_2_O_3_, soil	[292, 293]
NH_4_NO_3_ → N_2_O	Photochemical oxidation + reduction (denitrification)	Al_2_O_3_	[294]
NO_x_ → NO_3_^−^	Photochemical oxidation	TiO_2_	[295, 296]
NO_2_ → HONO, NO, N_2_O	Photochemical reduction	TiO_2_	[296]
NO_2_^−^ → NO_3_^−^	Photochemical oxidation	TiO_2_, ZnO, Fe_2_O_3_, WO_3_	[297]
NO_3_^−^ → NH_3_	Photochemical reduction	TiO_2_ plus electron acceptor	[298]
NO_3_^−^ or HNO_3_ → N_2_O, NO, HONO, NO_2_	Photochemical reduction (denitrification/renoxification)	Al_2_O_3_, TiO_2_, SiO_2_, α-Fe_2_O_3_, ZnO, CuCrO_2_, Na zeolite, sand Observed in snow	[299–305]
NO_3_^−^ → NO_2_^−^ (+ O_2_)	Photochemical reduction (+oxidation)	No facilitator Iron(III) oxide, soil, organic matter; TiO_2_ plus humic acids	[103, 306–309]
NO_2_ → HONO	Photochemical reduction	Humic acids, soot, soil Observed in ice	[157, 310, 311]
N_2_O → N_2_	Photochemical reduction	ZnO, Fe_2_O_3_, sand Humic and fulvic acids	[94, 95, 151, 312]
N_2_O → N_2_ + O_2_	Photochemical dissociation	ZnO, Cu(I) zeolites	[313, 314]
N_2_ → NH_3_	Photochemical reduction/(reductive) photochemical fixation	ZnO, Al_2_O_3_, Fe_2_O_3_, Ni_2_O_3_, CoO, CuO, Fe(III) in TiO_2_, Fe_2_O_3_-Fe_3_O_4_, MnO_2_, Sand, soil Aqueous suspensions of TiO_2_, ZnO, CdS, SrTiO_3_, Ti(III) zeolites Hydrous iron(III) oxide in the absence of O_2_	[2, 229, 315–321]
N_2_ + H_2_O → NH_3_ + O_2_	Photochemical reduction + oxidation	TiO_2_ in the absence of O_2_, α-Fe_2_O_3_, Fe(III)-doped TiO_2_	[58, 321, 322]
N_2_ → N_2_H_4_	Photochemical reduction	Sand	[2]
N_2_ + H_2_O → N_2_H_4_ + O_2_	Photochemical reduction + oxidation	TiO_2_ in the absence of O_2_	[322]
N_2_ + O_2_ → NO	Photochemical oxidation (oxidative) photochemical fixation	TiO_2_ in air	[323]
N_2_ → NO_2_^−^ N_2_ → NO_3_^−^	Photochemical oxidation (oxidative) photochemical fixation	Suspension of ZnO in the absence of O_2_ Aerated suspension of hydrous iron(III) oxide TiO_2_, soil	[320, 324, 325]
N_2_ + H_2_O → NO_2_^−^ + H_2_	Photochemical oxidation + reduction	ZnO-Fe_2_O_3_ under N_2_	[326]
*Metal compounds*			
Organic complexes of Fe, Al, Co, Ni (aq) → ionic Fe, Al, Co, Ni (aq)	Photochemical decomposition + decomplexation		[327, 328]
Organic complexes of Fe, Cu, Cr, Pb, V (aq) → colloidal Fe, Cu, Cr, Pb, V	Photochemical decomposition + precipitation		[328]
Organic matter (aq) + iron (aq) → organic matter + iron (s)	Photochemical flocculation		[193, 329]
FeOH^+^ (aq) → FeOOH	Photochemical oxidation		[330]
Fe(III) (hydr)oxides (s) → Fe(II) (aq)	(Reductive) photochemical dissolution of FeOOH + photochemical oxidation of organic matter (if present)	No facilitator Coprecipitated or dissolved organic matter, HSO_3_^−^, montmorillonite Accelerated in ice	[70, 71, 92, 122, 331–338]
Fe(II) (aq)/Fe(OH)_2_ + H_2_O → Fe(III) + H_2_	Photochemical oxidation + reduction	No facilitator Chromophores such as chlorophyll	[339, 340]
Fe(III)-carboxylate complexes (aq) → Fe(II) (aq)	Photochemical reduction + decomplexation		[66, 70, 341, 342]
Mn(IV) oxide → Mn(II) (aq)	(Reductive) photochemical dissolution	Dissolved organic matter Accelerated in ice	[337, 343–347]
Mn(II) (aq) → MnO_x_ (x = 1 to 2)	Photochemical oxidation	Organic matter, TiO_2_	[348, 349]
Cu(II) (aq) → Cu(I)	Photochemical reduction	Amino acids	[224, 225]
Cr(VI) (aq) → Cr(III) (aq)	Photochemical reduction	Ferritin, phenol	[350, 351]
ZnS + H_2_O → H_2_S → H_2_	Photochemical reduction + dissolution		[21, 251]
ZnS → Zn(0) + S(0)	Photochemical oxidation + reduction		[21]
CdS → Cd(II) + S(0)	Photochemical oxidation		[211]
HgS → Hg(II) (aq) + H_2_S	Photochemical dissolution		[228, 352]
HgS → Hg(0) + S(0)	Photochemical oxidation + reduction	Cl^−^	[25]
HgS → [Hg_2_Cl_2_ and other intermediates] → HgCl_2_	Photochemical oxidation, reduction/photochemical dissolution	Cl^−^	[25]
Hg(0) (aq) → Hg(II) (aq)	Photochemical oxidation		[352, 353]
Hg(II) (aq) → Hg(0) (aq)	photochemical reduction	Fe(III) species, TiO_2_, organic matter Observed in freshwater, seawater, and snow	[352, 354–357]
Hg(II) (aq) → HgCH_3_^+^	Photochemical methylation		[358]
HgCH_3_^+^ → Hg(II)	Photochemical demethylation		[359, 360]
HgCH_3_Cl → Hg(II) + Hg(0) + CHCl_3_ + HCHO	Photochemical demethylation + reduction		[361]
*Other elements*			
Plant material → H_2_	(Reductive) photochemical decomposition		[362, 363]
Dissolved organic P → inorganic phosphate	Photochemical decomposition (mineralization)		[364]
Phosphate adsorbed to Fe(III) oxides or Fe(III)-organic matter complexes → free phosphate	Photochemical desorption		[161, 365, 366]
HS^−^/S^2−^ → H_2_	Photochemical reduction	CdS, α-Fe_2_O_3_	[367, 368]
SO_2_ → SO_4_^2−^	Photochemical oxidation	TiO_2_, Fe_2_O_3_, ZnO, CdS	[369–372]
Thiols and SO_3_^2−^ → oxidized products	Photochemical oxidation	Ferritin	[255]
Alkyl sulfides + NO_x_ → aldehydes, sulfonic acids, SO_2_, SO_4_^2−^	Photochemical oxidation		[373]
O_2_ → H_2_O_2_	Photochemical reduction	ZnO, TiO_2_, sand in the presence of organic electron donors Aqueous Fe(III)-carboxylic acid complexes Tryptophan and tyrosine Porphyrins and phthalocyanines Algae (live or dead)	[34, 107, 246, 298, 374 *–* 376]
O_2_ → H_2_O	Photochemical reduction	α-Fe_2_O_3_ Dissolved Fe and humic substances (a catalytic cycle)	[123, 377]
H_2_O → H_2_	Photochemical reduction	Numerous catalysts, usually in the absence of O_2_, e.g., TiO_2_, ZnS, α-Fe_2_O_3_, hydrated Cu_2_O, tungstosilicate on TiO_2_, Ti(III)-zeolite, graphite oxide	[21, 22, 262, 315, 377–382]
H_2_O → O_2_	Photochemical oxidation	α-Fe_2_O_3_ + Fe(III) (aq), BiVO_4_ + electron acceptor, Mn_2_O_3_, λ-MnO_2_, Mn_3_O_4_, Co_3_O_4_ + sensitizer, AgCl, layered double hydroxide minerals Fe(OH)^2+^ (aq)	[383–390]
H_2_O → H_2_ + O_2_	Photochemical water splitting (oxidation + reduction)	TiO_2_, Fe_2_O_3_-Fe_3_O_4_, Fe_2_O_3_-FeS_2_, Cu_2_O, ZrO_2_, Ag zeolite, diverse two-mineral systems	[60, 137, 321, 322, 391–393]
As(III) (aq) → As(V) (aq)	Photochemical oxidation	No facilitator Ferrihydrite, kaolinite	[158, 394, 395]
As_4_S_4_ → As_4_S_4_ (polymorph)	Photochemical structural (crystal) modification		[396]
As_2_S_3_ → [As + S] + O_2_ → As_2_O_3_ As_4_S_4_ → As_2_O_3_	Photochemical oxidation/dissolution	Water	[396, 397]
Volatile organic compounds + NO_x_ → O_3_	Photochemical oxidation		[398]
Cl^−^ → Cl^−^_2_ (dichloride radical anion)	Photochemical oxidation	Chlorophyll, Hg(II)	[65, 352]
Cl^−^ + O_3_ → Cl_2_	Photochemical oxidation		[399]
NO_3_^−^ + Br^−^ → Br_2_	Photochemical oxidation		[400]

A suggested descriptor is given for each reaction as well as substances reported to facilitate the reaction (if any) and some relevant notes. These facilitating substances also occur naturally, or (in just a few instances) are reasonably similar to something that might occur naturally. About 15% of the studies cited here can be considered field studies, which means that a reaction was observed with both natural sunlight and natural substances as well as under representative environmental conditions, as opposed to the use of artificial light and/or laboratory-prepared equivalents of natural compounds

*Note on terminology* The term “photochemical” can be used to maintain a clear distinction between abiotic photoreactions and analogous reactions involving light and living organisms (phototrophy). For example, “iron(II) photooxidation” can refer to either a biological process driven by light (photobiological/phototrophic iron(II) oxidation) or a strictly chemical, abiotic process (photochemical iron(II) oxidation). Similarly, an abiotic process that converts water to O_2_ under the action of light may be described as “photochemical oxidation of water” rather than simply “photooxidation of water” (even though the latter is shorter and often understood to mean a photochemical reaction); this distinguishes it from light-induced biological oxidation of water that might occur simultaneously in the same environment

## Classification of photogeochemical reactions

**Fig. 2:**
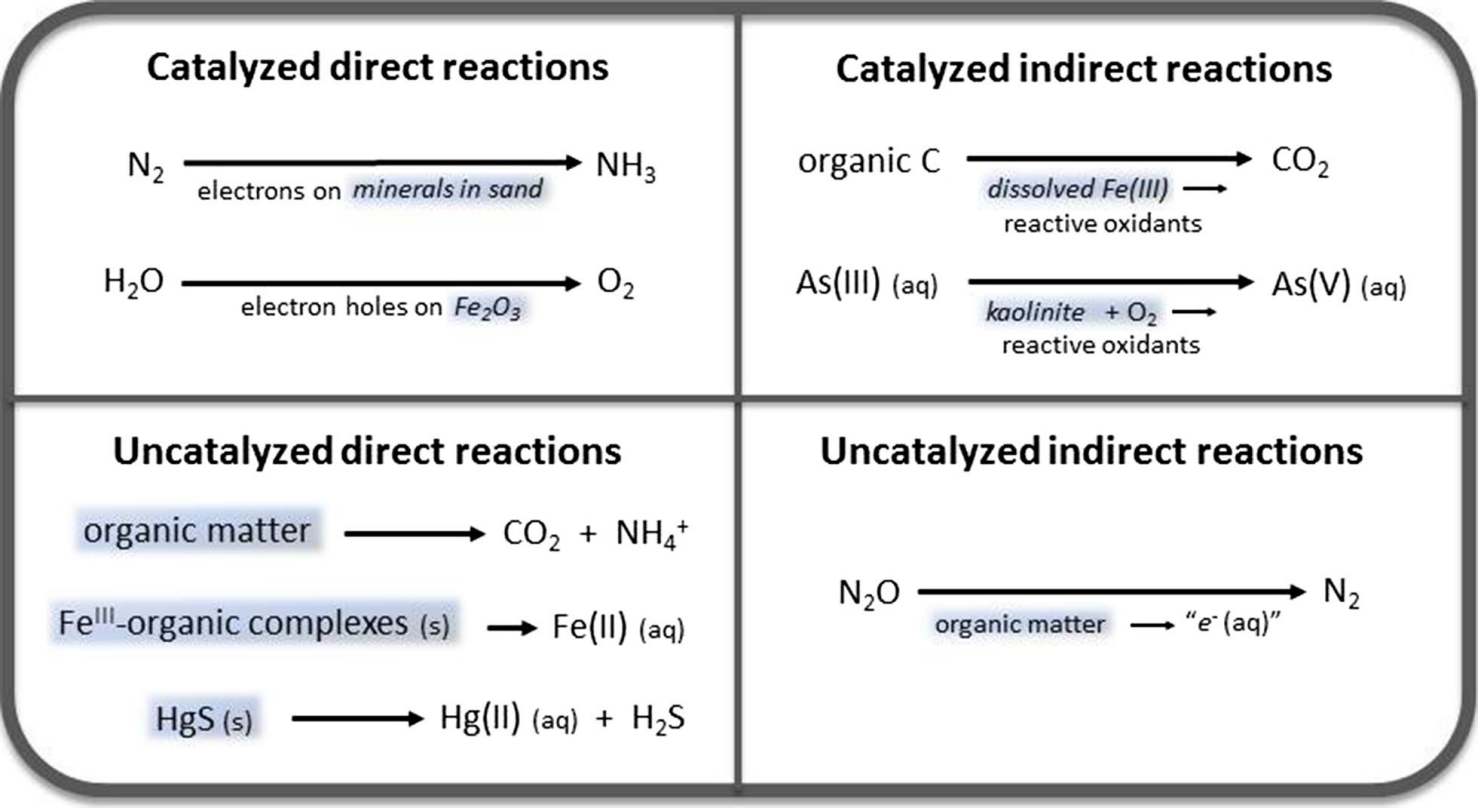
Photogeochemical reactions, if enough information is known, can be classified using general principles of photochemistry. Examples are given for each of four categories in a simple scheme of classification based on the mechanism of reaction. Light-absorbing materials are shaded and catalysts are shown in *italics*. Intermediate processes in indirect reactions are indicated as separate reactions below the main reaction *arrow*. For additional explanation of these mechanisms, see the text and the references for specific reactions listed in Table 1

**Fig. 3:**
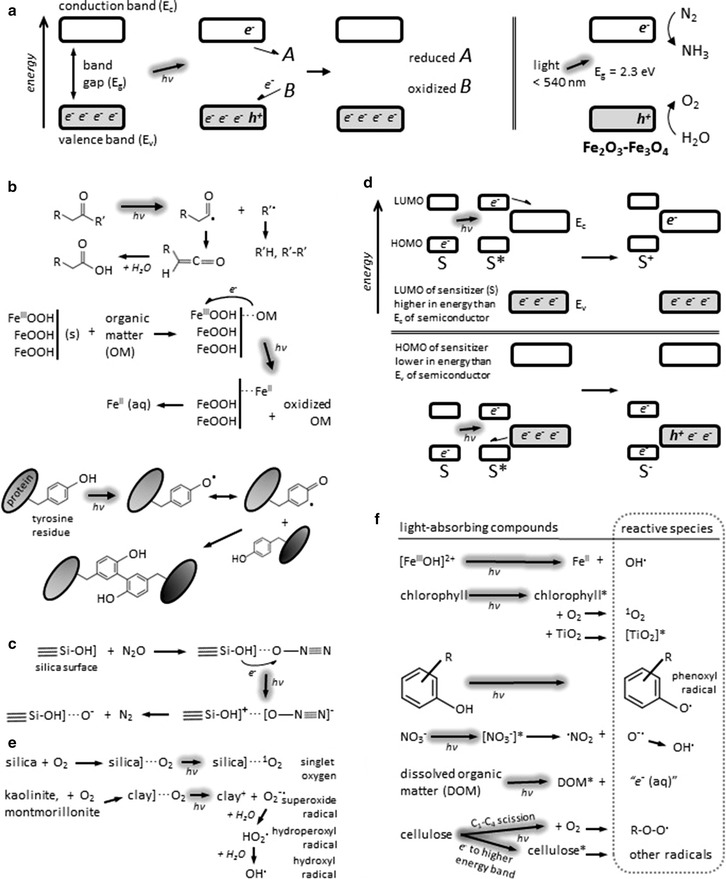
Simplified representations and some examples of processes that occur in photochemical reactions of natural substances: **a** promotion of electrons (*e* ^−^) and generation of electron vacancies (holes, *h* ^+^) upon irradiation of a semiconductor, which may then reduce and oxidize other substances; **b** excitement of organic compounds by sunlight which then directly react with other substances or are themselves altered, with examples of photochemical acidification, dissolution, and crosslinking; **c** photocatalysis via surface adsorption, which makes a species, here N_2_O, susceptible to the effect of light; **d** indirect generation, via a photosensitizer, of electrons and holes in a semiconductor: the difference between the highest occupied molecular orbital (HOMO) of the sensitizer and its lowest unoccupied molecular orbital (LUMO) is smaller than the band gap of the semiconductor, and therefore less energy is required to excite the sensitizer; **e** cooperative generation of transient reactive species by compounds that do not individually absorb sunlight; **f** generation of transient reactive species by light-absorbing compounds. *Arrows* with *shadows* indicate reactions induced by light (*hν*), *asterisks* (*) indicate excited species (electrons promoted to higher energy levels), *single brackets* (]) indicate mineral surfaces, and *dotted lines* (^…^) indicate surface adsorption. The references cited in the text offer additional, detailed explanations of these processes

The same principles that form the foundation of photochemistry can also be used to describe and explain photogeochemical reactions. If specific reactions are known, they may be distinguished as either photosynthetic reactions, photocatalytic reactions, or uncatalyzed reactions. In the most general sense, photosynthesis refers to any photochemical reaction for which the change in energy (ΔG) is positive. The energy of the products is greater than that of the reactants, and therefore the reaction is thermodynamically unfavorable, except through the action of light in conjunction with a catalyst [36] or a chromophoric system, for example, that mimics what occurs in plants [37]. Examples of photosynthetic reactions include the production of H_2_ and O_2_ from water and the reaction of CO_2_ and water to form O_2_ and reduced carbon compounds such as methane and methanol. Photocatalysis refers to photochemical reactions, accelerated by the presence of a catalyst, that have a negative change in energy and are therefore thermodynamically favored [36], such as the reaction of organic compounds with O_2_ to form CO_2_ and water. Finally, uncatalyzed photoreactions proceed through the action of light alone. For example, many organic compounds absorb light and suffer decomposition as a result. Figure 2 depicts a simple scheme for classifying photoreactions based on the requirement for a catalyst and whether a reaction proceeds by a direct or indirect mechanism, as further described below. Figure 3 shows some of the processes that operate in these reactions, also discussed below.

### Catalysis

A catalyst is a substance that increases the rate of a chemical reaction due to a change in mechanism, but does not experience any net change itself during the course of the reaction [37, 38]. A photocatalyst does this by absorbing light, but as described below, other substances that do not absorb light may nevertheless catalyze light-induced reactions. Strictly speaking, the term catalysis should not be used unless it can be shown that the number of product molecules produced per number of active sites on a substance (the turnover number) is greater than one [39]; this is difficult to do in practice, although it is often assumed to be true if there is no loss in the activity of the substance for an extended period of time [36]. Reactions which are not definitively catalytic may be designated as assisted photoreactions [36, 38] or photosensitized reactions. Photosensitized reactions involve transfer of energy from a light-absorbing species (photosensitizer) to another, nonabsorbing species, and therefore facilitate reaction of this nonabsorbing species [40]. If the photosensitizer remains intact it is effectively a photocatalyst. Furthermore, a substance may initially act as a photocatalyst in a reaction even if it eventually suffers light-induced decomposition. Descriptors such as those given here are most applicable when all of the participants in a specific reaction can be identified, not just individual reactants or products. In contrast, it is hard to classify observations in complex matrices such as soil if the complete reactions responsible for the observations are not first discerned.

### Direct reactions

Photochemical reactions can be further categorized as either direct or indirect. Direct reactions involve the substance that initially absorbs light [41–43] which reacts with other substances or is itself changed. Many photochemical reactions on Earth may be directly mediated by naturally occurring semiconductors that absorb ultraviolet and visible radiation. These are mostly transition metal oxides and sulfides and include abundant, widely distributed minerals such as hematite (Fe_2_O_3_), magnetite (Fe_3_O_4_), goethite and lepidocrocite (FeOOH), anatase and rutile (TiO_2_), pyrolusite (MnO_2_), pyrite (FeS_2_) chalcopyrite (CuFeS_2_), and sphalerite (ZnS) [44, 45]. Other types of minerals are also known to absorb light and directly participate in photoreactions, including silicates such as Ag_6_Si_2_O_7_ [46] and phosphates such as Cu_2_(OH)PO_4_ [47]. Light of energy equal to or greater than the band gap of a semiconductor is sufficient to promote electrons from the valence band to a higher energy level in the conduction band, leaving behind electron vacancies or holes (Fig. 3a). The excited electron and hole in the semiconductor can then, respectively, reduce and oxidize other compounds having appropriate redox potentials relative to the potentials of the valence and conduction bands [48]. The band gaps and absolute energy levels of many minerals are suitable, in theory, for a diverse array of photoreactions at interfaces with water, gases, and other solids. Naturally occurring semiconductors are almost exclusively inorganic compounds, with notable exceptions (notable because they occur widely) being melanin [49] and possibly cellulose [50, 51] and peptides [52–54].

Natural semiconducting minerals, like most minerals, are rarely pure; additional metals are almost always present [44], and these substitutional impurities can cause changes in energy levels and conductivity [44, 55]. Such alterations are manifested in photocatalytic activity. For example, the band gap of TiO_2_ decreases due to Fe impurities [56, 57], which extends its response to a wider range of solar radiation compared to pure TiO_2_; the efficiencies of photochemical oxidation and reduction reactions of TiO_2_ are also greater if Fe impurities are present [57, 58]. Similarly, the presence of Ti or V in magnetite enhances its photocatalytic activity relative to pure magnetite [59]. In addition to atoms of foreign elements, another common “defect” in minerals is deviation from stoichiometry due to vacancies (missing atoms), and this can also affect photochemical properties. For example, sulfur deficiencies in ZnS crystals impart increased photocatalytic activity under visible light to a material that normally absorbs little or no visible light [23]. In addition to chemical alterations, the photocatalytic activity of materials like these is also influenced by physical properties such as crystal structure and specific surface area [23, 56, 60].

Like inorganic minerals, many natural organic compounds also absorb sunlight and can react directly with other compounds or undergo reactions themselves (Fig. 3b); these include dissolved organic matter [61–63], “bioorganic” substances [64], chlorophyll [65], atmospheric humic-like substances [42], and soot or black carbon [42, 66]. Moreover, two species may combine to form a new species with even greater propensity to undergo direct photoreactions, as is often the case with intramolecular or intermolecular charge-transfer complexes among components of organic matter [67] or between transition metals and organic matter [68]. Sometimes this even leads to catalytic or autocatalytic cycles [69–71].

Finally, materials that do not absorb sunlight, such as silica, may nonetheless enable direct photoreactions. These materials are usually catalysts and act primarily via surface adsorption, which can alter the bond lengths and energies of a substance when it is bound to the catalyst [72, 73] and consequently alter the amount or wavelengths of sunlight absorbed by this substance [74, 75]. The bound substance then becomes susceptible to photolysis and other photoreactions (Fig. 3c). Depending on the nature of a substance, however, adsorption onto materials such as clay and ash can sometimes impede rather than facilitate photoreactions [76–78].

### Indirect reactions

Indirect photochemical reactions are initiated by substances that absorb radiation and subsequently facilitate other reactions that do not involve the original light-absorbing substance [42]. For example, excited electrons and holes can be indirectly generated in semiconductors by light of lower energy than the band gap: the semiconductor itself does not absorb this light, but another substance (possibly even another semiconductor) that does absorb this light may be excited, and if this substance is in contact with the semiconductor and has appropriate energy levels, electrons can then be transferred between the excited substance and the semiconductor [48, 68, 79–81] (Fig. 3d). The semiconductor, now carrying additional electrons or holes, can participate in redox reactions that would not otherwise occur. For example, TiO_2_ has a large band gap and is not normally excited by visible light; however, organic matter and natural chlorophyll derivatives are excited upon absorption of visible light, and in proximity to TiO_2_ can transfer electrons to TiO_2_ [82, 83]. This process is called charge injection, and is an example of photosensitization—reactions of TiO_2_ with additional substances are facilitated by the initial presence of organic matter or chlorophyll derivatives.

A substance may also participate indirectly in photochemical reactions by generating reactive species upon irradiation; these reactive species then engage in other reactions that do not involve the original light-absorbing substance [42]. For example, some aluminosilicates (e.g., zeolites) and non-transition-metal oxides (e.g., SiO_2_, Al_2_O_3_, MgO) can react with the oxygen in air upon irradiation to produce reactive oxygen species (ROS) such as singlet oxygen and superoxide [84, 85]. Photodegradation of an organic compound was observed in the presence of kaolinite and montmorillonite, for example, and was attributed to the formation of ROS on the surface of these minerals in the presence of molecular oxygen and water [86]. Since the organic compound in question does not absorb sunlight and the ROS are produced in a separate reaction, this is an indirect photoreaction, facilitated by the clay minerals which presumably act as catalysts by generating ROS from O_2_ upon exposure to light (Fig. 3e).

Along with minerals [87], other substances can indirectly facilitate photoreactions by generating reactive species in sunlight: dissolved and particulate organic matter [88–95], dissolved organic matter and silicate minerals in synergy [63], cellulose [50, 96, 97], lignin [98, 99], leaves of phototoxic plants [100], chlorophyll [101], nitrite and nitrate [102–104], flavins [41, 105], tryptophan and tyrosine [99, 106, 107], and aqueous iron(III) species [108–110]. In contrast to the typically strong oxidizing action of ROS, a strongly reducing species can also be generated which is usually represented as *e* ^−^ (aq), a hydrated electron, although its true nature and features are not completely understood. Hydrated electrons are evident upon irradiation of dissolved organic matter, for example [94, 95]. As might be expected, reactive species are formed on exposed soil surfaces [111, 112]; both the mineral and organic components of soil contribute to this process [113]. Indirect photolysis of organic compounds in soil has been observed to occur at depths of up to 2 mm due to migration of reactive species; in contrast, direct photolysis (in which the degraded compound itself absorbs light) is restricted to a photic depth of about ten times less [114, 115]. Both light penetration and transport processes such as diffusion influence the extent to which compounds are degraded by light in soil and similar media [116]. Indirect processes may operate during photodegradation of plant material as well [117]. In certain instances, however, the same substances listed above may also inhibit the formation of reactive species and therefore retard indirect photoreactions, as observed for chlorophyll [118], carotenoids [119], and organic matter in soil and water [76, 120].

## Experimental approaches

**Fig. 4:**
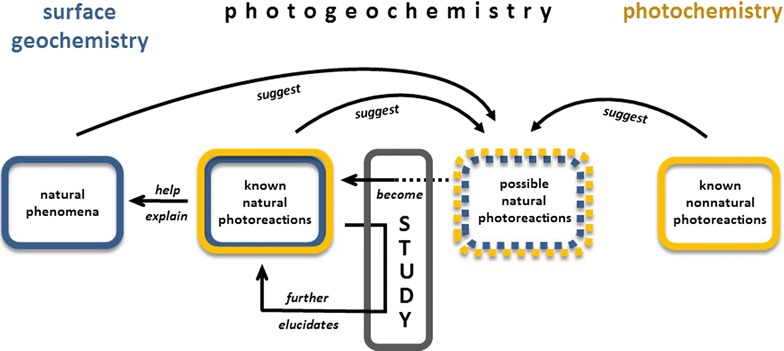
The study of photogeochemistry reflects the overlap between surface geochemistry and photochemistry. The *curved arrows* represent three different but complementary approaches which can lead to the discovery of natural photoreactions: observing natural phenomena, extending known natural photoreactions, and contextualizing photoreactions that are not known to occur naturally

Studies in photogeochemistry may take several different paths, depending on the source of inspiration for identifying and investigating natural photochemical reactions (Fig. 4). Oftentimes photogeochemistry distinctly parallels biogeochemistry. As mentioned above, early research sometimes intentionally used biological phenomena as a starting point to search for analogous photochemical reactions. Other studies simply explored the effect of light on different materials, and as a result also discovered photochemical reactions analogous to biological processes. Photochemical counterparts have since been confirmed for many well-known biochemical reactions. These include photochemical disproportionation of acetic acid [121, 122] which is analogous to acetoclastic methanogenesis, and light-induced depletion of O_2_ via a catalytic cycle involving iron and organic matter [123], analogous to consumption of O_2_ by microorganisms. Estimates of the environmental significance of photochemical reactions relative to biological reactions have been offered on occasion, as for photochemical production of gases from plant litter [124, 125], and the photofixation of N_2_ in deserts, estimated as 20 kg N ha^−1^ year^−1^, which is about one third of that fixed by lightning and about 10% of that fixed biologically on Earth [126]. In contrast to these processes, in which biological reactions predominate (at least on a global level), the rate of degradation of dissolved lignin in rivers by photochemical mechanisms was found to be several times larger than by biological mechanisms [127]. Witz, based on his (nonbiological) studies with cellulose and other plant fibers [14], concluded that light is indeed an integral participant in natural decomposition: “In nature, once the life of plants is extinguished, cellulosic matter and other structured matter must no doubt pass progressively under the influence of light, air, and humidity … and are eventually transformed into gaseous compounds and colored humic materials.”

### Extension of known photoreactions

The most obvious experimental precedent in photogeochemistry is a natural photoreaction that has already been ascertained. Known reactions may be further investigated as to their context, mechanisms, and environmental significance. For example, the greenhouse gases CO_2_, CH_4_, and N_2_O are the subject of a large amount of ongoing interdisciplinary research. Natural production and consumption of these gases at the earth’s surface are ascribed largely to biological activity [128–131], which remains the focus of most research, in spite of studies that have demonstrated photochemical production and consumption (see Table 1). Similarly, mineralization of organic carbon, nitrogen, and phosphorus in soil and water, the biological drivers of which are extensively studied, may also proceed photochemically. It is interesting to note that biologically recalcitrant portions of organic matter can be quite susceptible to photodegradation [132, 133]; the consequent release of labile organic and inorganic compounds can stimulate biological activity [134–136].

Sometimes a particular reaction, when placed in a certain environmental context, may even affect existing paradigms. For example, it is generally (and logically) assumed that in water classified as anoxic there can be no reactions involving molecular oxygen, including aerobic metabolism. However, some naturally occurring minerals are known to facilitate the photochemical oxidation of water to molecular oxygen; such “photochemical sources of oxidizing power in low-oxygen environments” [137] may be active alongside or in place of other sources of oxygen such as air or photosynthetic organisms. Similarly, organic acids known to be produced during the photodecomposition of organic matter may form a connection between light exposure and soil acidity, a simple but unestablished possibility next to the usual factors that determine soil pH.

While investigation of known natural photoreactions can be extended by pursuing additional work with the same substances, knowledge of natural photoreactions may also support inquiry into photoreactions of distinct but related substances. For example, the susceptibility to photodegradation of polycyclic aromatic hydrocarbons and related condensed aromatic compounds has been reported [e.g., 78, 138–140]. These studies focus on relatively simple molecules which are either regarded as naturally occurring pollutants or are components of dissolved organic matter. At the same time, the incomplete combustion of natural organic materials leaves solid residues (“charcoal”, “biochar”, or “pyrogenic black carbon”) that contain analogous extended aromatic structure [141–143]. It may therefore be suggested that this ubiquitous material, commonly deemed environmentally persistent [63, 140, 143, 144] and therefore paradoxical (since it does not accumulate in the environment) [145, 146], is also degraded upon exposure to sunlight.

The study of photogeochemistry, while purely chemical in nature, may even venture into the domain of biology and identify more of the ways in which compounds derived from living organisms can influence abiotic photochemistry [e.g., 81], as well as more of the unique relationships between photochemical reactions and biological metabolism known as photobiocatalysis [147–149].

### Observation of natural phenomena

Specific photoreactions are often planned and conveniently observed in the laboratory, using artificial light sources or sunlight itself, where it is easy to confirm the identity of the substances involved, design reaction vessels, characterize the light, and adjust the reaction environment. However, observations of natural phenomena can offer opportunities to consider unknown photochemical reactions possibly associated with these phenomena. For example, by the 1970s it was generally agreed that nitrous oxide (N_2_O) has a short residence time in the troposphere, although the explanation for its removal was incomplete. Since N_2_O does not absorb light of wavelengths greater than 290 nm, direct photolysis had been discarded as a possible explanation. It was then observed that light would decompose chloromethanes when they were adsorbed on silica sand [150], and this occurred at lower energies (longer wavelengths) than the absorption spectra for the free compounds. The same phenomenon was observed for N_2_O on natural sand, leading to the conclusion that particulate matter in the atmosphere is responsible for the destruction of N_2_O via surface-sensitized photolysis [151]. Indeed, the idea of such a sink for atmospheric N_2_O was supported by reports of low concentrations of N_2_O in the air above deserts, where there is a large amount of suspended particulate matter [152]. In general, simple atmospheric gases (e.g., CO_2_, CO, CH_4_, N_2_O, N_2_, H_2_O, H_2_, O_2_) do not absorb ultraviolet and visible sunlight at the earth’s surface, and the cooperation of particulate matter is necessary for photoreactions involving these gases; such reactions are therefore heterogeneous. Other gases, however, such as some of the volatile compounds emitted from living plants [153, 154], burning plants [155] and soils [156], do absorb sunlight and can undergo homogeneous as well as heterogeneous reactions.

As another example, the observation that the amount of nitrous acid in the atmosphere greatly increases during the day led to insight into the surface photochemistry of humic acids and soils and an explanation for the original observation [157]. Fluctuations such as this are often a clue to the existence of photochemical reactions, which operate only during the day. Diurnal photogeochemical cycles often have a significant influence on the amounts of redox-sensitive elements in aqueous environments [70, 158–160]. Furthermore, multiple elemental cycles can be linked via photoreactions that directly affect both elements, as occurs during the concurrent oxidation of organic matter and reduction of iron [92]. The effect of light on one element can also indirectly affect other elements: a daily cycle of photoreduction, reoxidation, and precipitation of iron(III) species affects dissolved As, Cu, and P, which adsorb to iron(III) oxides as they reappear at night and may be subsequently released the next day upon photoreduction of the same iron oxides [158, 159, 161].

### Contextualization of nonnatural photoreactions

Although photogeochemistry describes reactions among substances known to occur naturally, studies of similar substances may nonetheless point towards greater understanding of natural processes. A general example demonstrates this: it has been shown that samples of clay minerals found in soils can accelerate the photodegradation of synthetic chemicals via production of reactive oxygen species [e.g., 86]; it may therefore be assumed that many naturally occurring compounds are similarly affected. The conversion of N_2_ to NH_3_ and NO_3_^−^ has been observed upon irradiation with visible light in the presence of Fe_2_Ti_2_O_7_ [162, 163]. While such a compound is not known to occur naturally, it is related to known minerals like ilmenite (FeTiO_3_), ulvospinel (Fe_2_TiO_4_), pseudorutile (Fe_2_Ti_3_O_9_), and various titanium-substituted iron oxides, and can form when ilmenite is heated [162, 164]; these naturally occurring minerals might therefore also react with N_2_ under certain conditions.

## Outlook

Principles of photochemistry can be readily merged with geochemistry in investigation as well as education. Given the broad response of natural substances to light, recognizing photochemical reactions in the environment is part of understanding its fabric of interconnected processes, particularly on land, where this has not been explored as much as in water or the atmosphere. As remarked by Formenti and Teichner [40] concerning heterogeneous photochemistry, “there are so many different possibilities”, an outlook reiterated by Cooper and Herr [165] for aqueous photochemistry which is easily extended to photogeochemistry: “there are a seemingly endless number of combinations and permutations to study.” This does not enjoin an unattainable research agenda, but rather affirms ample opportunity for geoscientists to incline their curiosity toward what happens on Earth when the sun appears.
